# NK Cells and Innate-Like T Cells After Autologous Hematopoietic Stem Cell Transplantation in Multiple Sclerosis

**DOI:** 10.3389/fimmu.2021.794077

**Published:** 2021-12-16

**Authors:** Josefine Ruder, Jordan Rex, Simon Obahor, María José Docampo, Antonia M. S. Müller, Urs Schanz, Ilijas Jelcic, Roland Martin

**Affiliations:** ^1^ Neuroimmunology and Multiple Sclerosis (MS) Research Section (NIMS), Department of Neurology, University and University Hospital Zurich, Zurich, Switzerland; ^2^ Department of Medical Oncology and Hematology, University and University Hospital Zurich, Zurich, Switzerland

**Keywords:** aHSCT, multiple sclerosis, innate-like T cells, NK cells, CD56bright NK cells, atypical T cells, MAIT cells, NKT cells

## Abstract

Multiple sclerosis (MS) is an autoimmune disease of the central nervous system, in which autoreactive T and B cells play important roles. Other lymphocytes such as NK cells and innate-like T cells appear to be involved as well. To name a few examples, CD56^bright^ NK cells were described as an immunoregulatory NK cell subset in MS while innate-like T cells in MS were described in brain lesions and with proinflammatory signatures. Autologous hematopoietic stem cell transplantation (aHSCT) is a procedure used to treat MS. This procedure includes hematopoietic stem/progenitor cell (HSPC) mobilization, then high-dose chemotherapy combined with anti-thymocyte globulin (ATG) and subsequent infusion of the patients own HSPCs to reconstitute a functional immune system. aHSCT inhibits MS disease activity very effectively and for long time, presumably due to elimination of autoreactive T cells. Here, we performed multidimensional flow cytometry experiments in peripheral blood lymphocytes of 27 MS patients before and after aHSCT to address its potential influence on NK and innate-like T cells. After aHSCT, the relative frequency and absolute numbers of CD56^bright^ NK cells rise above pre-aHSCT levels while all studied innate-like T cell populations decrease. Hence, our data support an enhanced immune regulation by CD56^bright^ NK cells and the efficient reduction of proinflammatory innate-like T cells by aHSCT in MS. These observations contribute to our current understanding of the immunological effects of aHSCT in MS.

## Introduction

Over 2.5 million people worldwide are affected with multiple sclerosis (MS), a chronic, demyelinating disease of the central nervous system (CNS), which is usually diagnosed at the young age of 20-40 years. Autoimmune inflammation is considered the main pathomechanism, which results in demyelination, neurodegeneration, glia activation and metabolic changes, the latter being more pronounced in progressive MS forms (1). A large body of evidence supports the importance of T cells in the pathogenesis of MS, especially of CD4+ T cells (1). In addition, recent data have shown that B lymphocytes, as another adaptive immune cell type, also play a crucial role (2–4).

Besides adaptive immune cells, available data indicate that innate immune cells including innate lymphoid cells (ILCs) and innate-like T and B lymphocytes are involved in MS pathogenesis (5). Amongst ILCs, natural killer (NK) cells are most important to mention (5). NK cells are a source of immunoregulatory cytokines, they interact with other immune cells and can directly kill target cells. For instance, the killing of mutated tumour cells or virus-infected cells can be triggered *via* downregulation or absence of MHC (“missing self”), or also *via* antibody-dependent cellular cytotoxicity (ADCC) (6, 7). In the healthy condition, NK cell activation and killing is controlled by multiple NK receptors with a balance between activating receptors e.g. for Fc region of IgG leading to ADCC, and inhibitory receptors recognizing e.g. MHC, resulting in the attack of cells with low or absent MHC expression (6). Certain NK receptor variants are associated with decreased MS risk, highlighting the importance of NK cells and their receptors in MS pathophysiology (8). However, how NK cells are implicated in the pathophysiology of MS is not fully understood. Certain populations might enhance disease progression by harming the myelin sheath *via* ADCC, while others can have immunoregulatory effects like killing activated T cells (9).

In the innate-like lymphocyte compartment, cells that have been shown to be involved in MS are γδ T cells, NKT cells and mucosa-associated invariant T (MAIT) cells (5). All these innate-like T cells share certain features. Their T cell receptor (TCR) repertoire diversity is limited (10), they show antigen specificity against a restricted number of antigens, are not restricted by classical MHC molecules, mostly reside in barrier organs like mucosal tissues, and they are able to respond faster after activation compared to adaptive immune cells (11). Similar to conventional T cells, innate-like T cells mature in the thymus and undergo comparable positive and negative selection processes (12). Mechanisms by which they contribute to the inflammation in MS include the migration to the inflammatory lesions in the brain, the secretion of proinflammatory cytokines, thereby enhancing local inflammation, and possibly also cytotoxicity towards oligodendrocytes (5).

Despite enormous advances in the last 25 years, the treatments of MS are only partially effective and a fraction of MS patients still develops disability over time. Autologous hematopoietic stem cell transplantation (aHSCT) is a long-established procedure that, besides its broad use in haematological malignancies and as a rescue treatment after cancer chemotherapy, has been applied in the last 30 year for the treatment of autoimmune diseases. Among these, aHSCT is most frequently used for treating MS, and, based on rapidly growing data, it appears superior to even the most effective, approved therapies (13). The proposed mechanism(s) of action of aHSCT include, first, the depletion of autoreactive immune cells and the subsequent reconstitution of a “new” immune system from the re-infused autologous hematopoietic stem/progenitor cells (HSPCs) (13–18). Additionally, in the context of the profoundly lymphopenic environment shortly after the aHSCT, increased numbers and/or enhanced function of immunoregulatory cell populations have been described. Therefore, the generation of a “new” TCR repertoire and enhanced immune regulation are believed to be important aspects for the efficacy of aHSCT in MS (13–16).

Since innate immune cells have received relatively less attention than adaptive immune cells in the field of aHSCT in MS, we have studied here the effects of aHSCT on NK and innate-like T cells to assess their potential involvement in the beneficial effects of this treatment in MS.

## Material and Methods

### Patients and Samples

All MS patients eligible for transplantation were asked for written informed consent and included into the local aHSCT-in-MS registry. Ethic protocols for the collection of biomaterials and to study aHSCT are in place (BASEC-No. 2018-01854). Patients were treated with aHSCT because of (1.) failure of highly active disease-modifying therapy as evident by (2.a.) clinical activity (relapses), (2.b.) and/or radiological (new and/or contrast enhancing lesions) inflammatory activity, (2.c.) and/or clinical progression. Further limitations to receive aHSCT included (3.) age (18-55 years), (4.) disease duration (max. 15 years) and (5.) neurological disability status as measured by expanded disease status scale (EDSS; 2.0-6.5).

The conditioning regimen consisted of 4 g/m² cyclophosphamide and 30 Mio units/day granulocyte-colony stimulating factor (G-CSF, filgrastim). The graft containing the mobilized CD34+ HSPCs was collected by leukapheresis. The ablative conditioning followed the BEAM-ATG protocol. BEAM contains 300 mg/m² BCNU (carmustine) i.v. day -6, 200 mg/m² etoposide i.v. day -5 to -2, 200 mg/m² Ara-C (cytarabine) i.v. day -5 to -2, 140 mg/m² melphalan i.v. day -1. Day 0 is defined as the day of the re-infusion of the graft containing the autologous HSPCs (4-8 x 10^6^ CD34+ cells/kg body weight). For *in vivo* depletion of residual T cells both in the graft, but also in the patient, rabbit anti-thymocyte globulin (ATG) was given on day 1 and 2 at 3.75 mg/kg i.v.

**Figure 1:**
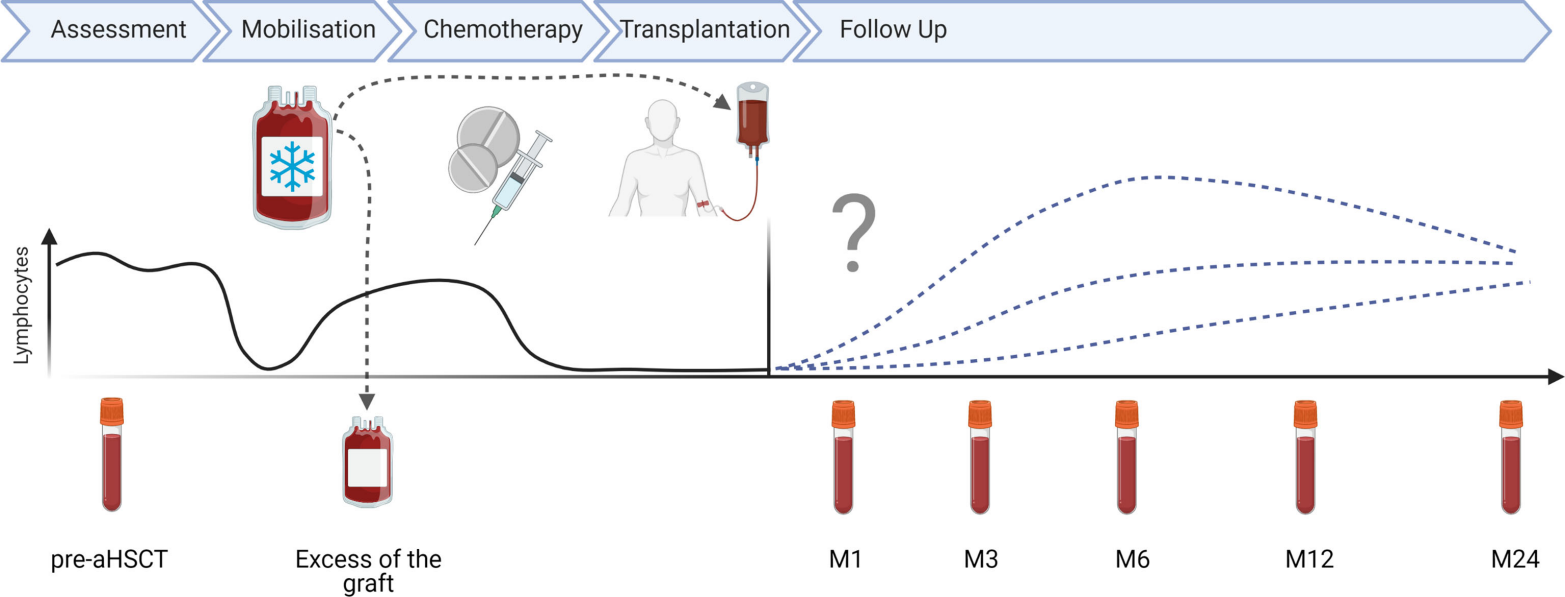
aHSCT in MS treatment scheme: schematic overview of aHSCT in MS procedure and sampling time points in months (M).

EDTA-anticoagulated blood was collected from MS patients before and at several time points after aHSCT (months 1, 3, 6, 12, 24). Additionally, excess material of the apheresis product for HSPC collection was examined. A schematic representation of the aHSCT procedure in MS and sampling times are depicted in **Figure 1**. Details about the 27 MS patients included in this cohort are included in **Table 1**. Blood from a cohort of 12 age- and sex-matched healthy controls (HCs) and 10 untreated relapsing remitting MS (RRMS) patients (**Table 1**), as well as three HC leukapheresis products without prior mobilization, and one anonymised buffy coat from the “Blood donation center Zurich SRK” were included as controls. **Table 2** depicts selected statistics about the cohort. Peripheral blood mononuclear cells (PBMCs) were freshly isolated from these materials using Ficoll-Paque™ density gradient. PBMCs were then cryopreserved for 24-48h at -80°C prior to long-term storage in liquid nitrogen, so all PBMCs underwent one single freeze/thaw cycle.

**Table 1:** Clinical and demographic characteristics of MS patients treated with aHSCT as well as healthy controls and RRMS disease controls.

	Donor	Age at treatment	Diagnose	Sex
aHSCT in MS	aHSCT_MS_01	38	RRMS	female
	aHSCT_MS_02	36	RRMS	male
	aHSCT_MS_03	53	PPMS	female
	aHSCT_MS_04	32	RRMS	female
	aHSCT_MS_05	47	PPMS	male
	aHSCT_MS_06	49	PPMS	female
	aHSCT_MS_07	33	SPMS	female
	aHSCT_MS_08	29	PPMS	male
	aHSCT_MS_09	48	SPMS	female
	aHSCT_MS_10	36	SPMS	female
	aHSCT_MS_11	47	RRMS	male
	aHSCT_MS_12	44	SPMS	male
	aHSCT_MS_13	41	PPMS	male
	aHSCT_MS_14	25	PPMS	male
	aHSCT_MS_15	53	SPMS	female
	aHSCT_MS_16	33	RRMS	female
	aHSCT_MS_17	39	RRMS	female
	aHSCT_MS_18	47	RRMS	male
	aHSCT_MS_19	43	PPMS	male
	aHSCT_MS_20	47	PPMS	male
	aHSCT_MS_21	25	RRMS	male
	aHSCT_MS_22	54	SPMS	female
	aHSCT_MS_23	43	RRMS	male
	aHSCT_MS_24	44	SPMS	female
	aHSCT_MS_25	44	SPMS	male
	aHSCT_MS_26	40	RRMS	female
	aHSCT_MS_27	51	SPMS	male
HC	HC_01	34	none	male
	HC_02	36	none	male
	HC_03	55	none	male
	HC_04	37	none	male
	HC_05	41	none	female
	HC_06	46	none	female
	HC_07	37	none	male
	HC_08	43	none	male
	HC_10	26	none	female
	HC_11	53	none	female
	HC_12	45	none	female
	HC_13	39	none	female
RRMS	RRMS_01	58	RRMS	female
	RRMS_02	35	RRMS	male
	RRMS_03	38	RRMS	female
	RRMS_04	37	RRMS	female
	RRMS_05	32	RRMS	female
	RRMS_06	34	RRMS	female
	RRMS_07	34	RRMS	male
	RRMS_08	23	RRMS	male
	RRMS_09	35	RRMS	male
	RRMS_10	43	RRMS	female

**Table 2:** Comparison of clinical and demographic characteristics between aHSCT and control groups.

	Age			Sex		Diagnosis		
	Mean	Min	Max	Female	Male	RRMS	SPMS	PPMS
aHSCT in MS	41.5	25	54	13 (48%)	14 (52%)	10 (37%)	9 (33%)	8 (30%)
HC	41.0	26	55	6 (50%)	6 (50%)	0 (0%)	0 (0%)	0 (0%)
RRMS	36.9	23	58	6 (60%)	4 (40%)	10 (100%)	0 (0%)	0 (0%)

T-test comparing the age of aHSCT vs. HC didn’t show a significant difference (p = 0.85). Chi-square test of independence comparing the sex ratio between aHSCT and HC didn’t show a significant difference (p = 1).

### Flow Cytometry

To immunophenotype the collected PBMCs, we developed three flow cytometry panels, a broad cell subset panel an NK and innate-like T cell panel and a CCR6 panel. For the broad cell subset panel and the CCR6 panel, samples from all 27 patients (pre = 26, M1 = 21, M3 = 19, M6 = 27, M12 = 25, M24 = 11), HC (= 12) and RRMS (= 10) were used, while 7 patients were used for the NK and innate-like T cell panel (patients no. 13, 17, 18, 19, 20, 24 and 26).

We used the following fluorophore-conjugated antibodies: anti-CD45 PerCP-Cy5.5, anti-CD3 BV786, anti-CD56 PE, anti-CD16 AF700, anti-Vα7.2 BV421, anti-Vδ2 BV711, anti-CD8 BV510 and anti-CCR6 BV785 (all from BioLegend^®^), anti-CD4 PE-Texas Red (Invitrogen™), anti-CD14 Pacific Blue, anti-TCRγδ FITC, anti-CD3 AF700 (all from BD) and anti-CD161 APC (Miltenyi Biotec). To exclude dead cells, we used the LIVE/DEAD™ fixable dyes Near-IR and Green (Invitrogen™). In brief, frozen PBMCs were thawed and counted. One million cells were first stained with the viability marker and blocked with purified human IgG (Sigma) and then stained with the respective surface markers. Samples were acquired with a BD LSR Fortessa flow cytometer and analysed using the software FlowJo (FlowJo LLC). Gating is shown in **Supplementary Figures 1–3**. Statistical tests included first a global test (ANOVA or Kruskal-Wallis) and in case of significance followed by pairwise comparisons (t-test or Wilcoxon). Post-aHSCT time points were usually compared to HC group whenever present, otherwise the pre-aHSCT time point was the reference group. Significance levels were ns for p > 0.05, * for p <= 0.05, ** for p <= 0.01, *** for p <= 0.001 and **** for p <= 0.0001. Statistical analysis as well as visualizations were performed in R Core Team (2020).

## Results

**Figure 2:**
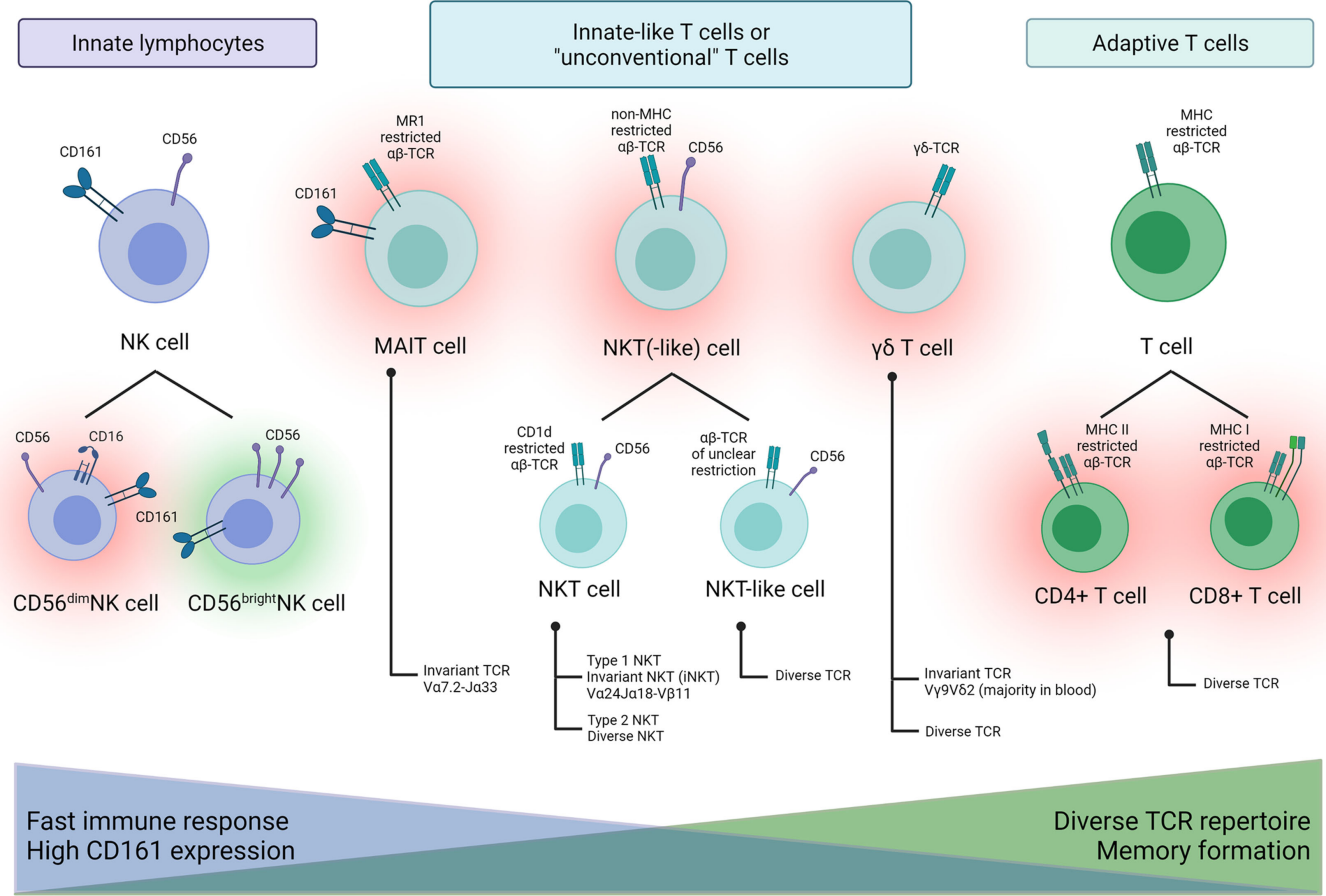
Schematic overview of NK and innate-like T cell populations characterized in this study and their possible proinflammatory (red halo) or beneficial (green halo) role in MS.

To characterize NK and innate-like T cells before and after aHSCT in MS patients, three flow cytometry panels were used. For an overview of the studied NK and innate-like T cell populations and their nomenclature see **Figure 2**. We are aware that this is a simplification. Nevertheless, it should help understanding the variety of innate immune cells that we studied and their potential role in aHSCT.

**Figure 3:**
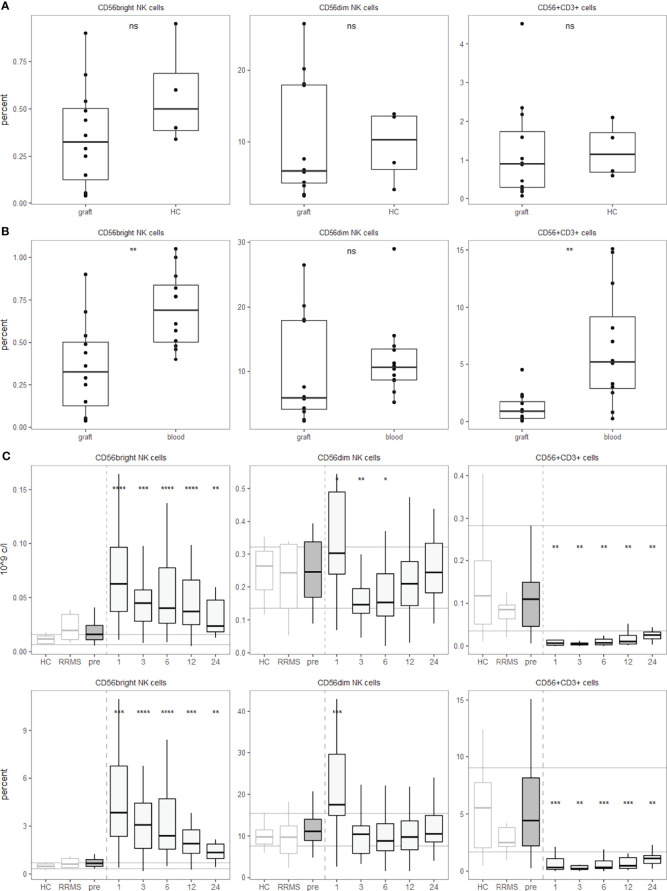
aHSCT but not the mobilization regimen is followed by a shift from CD56^dim^ towards CD56^bright^ NK cells and a strong decrease in innate-like CD56+CD3+ T cells. PBMCs were stained with surface fluorophore-conjugated antibodies and analyzed by flow cytometry. Percentages are fractions of total PBMCs and absolute cell numbers were calculated from lymphocyte numbers measured in clinical routine. **(A)** NK and innate-like T cell percentages in the graft after the mobilization regimen, but before aHSCT compared to three leukapheresis and one buffy coat. Number of samples were graft = 12, HC =4. **(B)** NK and innate-like T cell percentages in the graft after the mobilization regimen, but before aHSCT compared to the pre-aHSCT PBMCs of the respective patients. Number of samples were graft = 12, blood = 12. **(C)** Absolute (top) and relative (bottom) NK and innate-like T cell dynamics pre- and post-aHSCT. Horizontal lines reflect the median and 10^th^/90^th^ percentile of age- and sex-matched HCs. Number of samples were pre = 26, M1 = 21, M3 = 19, M6 = 27, M12 = 25, M24 = 11, HC = 12, RRMS = 10. As a global test, ANOVA was used, and in case of significance followed by pairwise comparisons (HC vs 1, 3, 6, 12 and 24 months) using t-test.

Within the hematopoietic system, the surface molecule CD56, also known as neural cell adhesion molecule (NCAM), in the absence of CD3 usually identifies NK cells (19). The degree of CD56 expression on NK cells subdivides them into a CD56^bright^ and a CD56^dim^ population. We first analysed PBMCs with a broad cell subset panel for major leucocyte subsets. Among NK cells, we differentiated CD56^bright^ and CD56^dim^ NK cells. Interestingly, the percentage of NK cells in the graft was not affected to a great extent by the mobilization regimen of cyclophosphamide and G-CSF (**Figure 3A**). However, compared to the matched pre-aHSCT sample, CD56^bright^ NK cells and CD56+CD3+ cells are slightly decreased (**Figure 3B**).

After BEAM-ATG and the transplantation, we observed a significant increase in the abundance of CD56^bright^ NK cells after aHSCT, while CD56^dim^ NK cells peaked one month post-aHSCT, then declined with slow recovery over the two years of observation (**Figure 3C**). Interestingly, older individuals and progressive patients recovered with higher percentages of NK cells of both CD56bright and CD56dim phenotype (**Supplementary Figures 4A, B**).

Expression of CD56 is not limited to NK cells, but also observed on certain T cells. A subset of myelin-reactive CD4+ T cells from MS patients expresses CD56 and can lyse target cells expressing CD56 by homotypic interactions (20). This mechanism could be involved in oligodendrocyte damage (21). In αβ T cells, expression of CD56 is mostly associated with CD8+ T cells, but CD4+ T cells as well as γδ T cells can also express this marker (19). Hence, CD3+CD56+ double positive cells are a population containing several innate-like T cell populations, but are not entirely composed of them. Most importantly, *bona fide* αβ TCR expressing activated myelin-specific CD4+ T cells can express CD56 (20). Interestingly, we saw a very efficient and highly significant depletion of CD56+CD3+ double positive cells below pre-aHSCT and HC levels for at least two years (**Figure 3C**), and this depletion was not observed to this extent after the mobilization regimen (**Figures 3A, B**).

These findings, together with studies showing an involvement of NK and innate-like T cells in MS, led us to examine these populations in the setting of aHSCT in MS. With a panel that allowed us to differentiate innate-like T cells like MAIT cells, NKT(-like) cells and γδ T cells from the innate NK cell subpopulations, we studied their immune reconstitution in MS patients post-aHSCT in seven individuals of our cohort.

**Figure 4:**
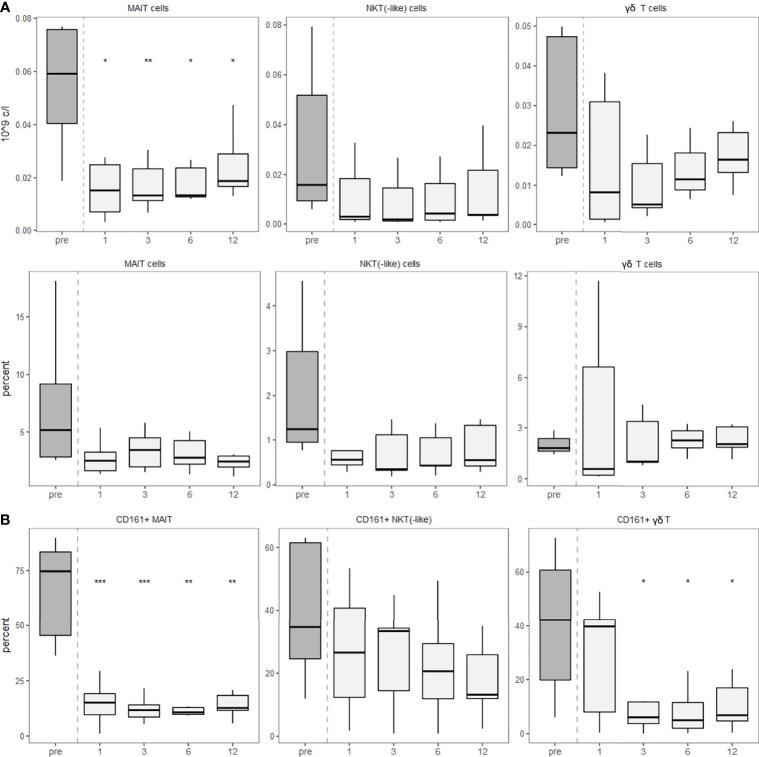
Innate-like T cells recover slowly after aHSCT in MS and show a lower expression of CD161. PBMCs were stained with fluorophore-conjugated surface antibodies and analyzed by flow cytometry. Percentages are fractions of total lymphocytes and absolute cell numbers were calculated from lymphocyte numbers measured in clinical routine. **(A)** Absolute (top) and relative (bottom) recovery of MAIT, NKT(-like) and γδ T cells post-aHSCT in MS. **(B)** Percentage of CD161-expressing cells within MAIT cells (left), percentage of CD161-expressing cells within NKT(-like) cells (middle) and percentage of CD161-expressing cells within γδ T cells (right). Number of samples were pre = 7, M1 = 7, M3 = 7, M6 = 7, M12 = 7. As a global test, Kruskal-Wallis was used, and in case of significance followed by pairwise comparisons (pre vs 1, 3, 6 and 12 months) using Wilcoxon signed-rank test.

We used CD3 in combination with the invariant TCR Vα7.2 to identify MAIT cells, the γδ TCR to identify γδ T cells and exclusion of these TCRs and positivity for CD56 and CD3 to identify NKT and NKT-like cells. NKT cells are identified by the restriction of their αβ TCR to CD1d, but beyond that, specific markers for NKT cells are lacking (22). For this reason, co-expression of CD3 and CD56 as NK cell marker are often used for their identification. The analysis of MAIT cells, γδ T cells and NKT(-like) cells showed that all of them decreased after aHSCT and remained depleted for at least one year (**Figure 4A**). To further examine innate-like T cells and their capacity to enter specific tissues, we studied tissue-homing receptors. First, we focused on the tissue-homing receptor CD161, which is expressed on NK cells but also on several T cell subsets (23). We found a lower percentage of innate-like T cells expressing CD161 post-aHSCT than pre-aHSCT (**Figure 4B**).

**Figure 5:**
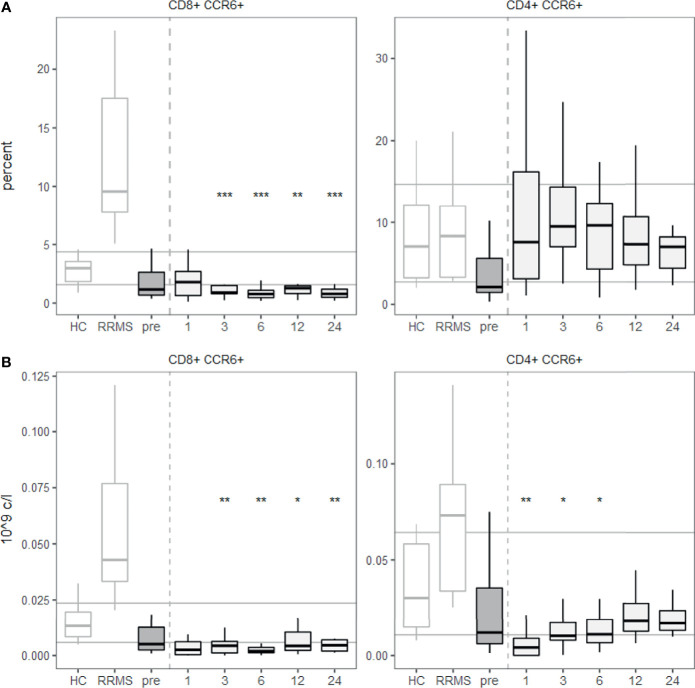
A lower percentage of CD8+ T cells express CCR6 from 3 months post aHSCT onwards. PBMCs were stained with fluorophore-conjugated surface antibodies and analyzed by flow cytometry. **(A)** Percentages are fractions of CD8+ (left) or CD4+ (right) T cells respectively. **(B)** Absolute number of CD8+ CCR6+ T cells (left) and CD4+ CCR6+ T cells (right). Number of samples were pre = 26, M1 = 21, M3 = 19, M6 = 27, M12 = 25, M24 = 11, HC = 12, RRMS = 10. As a global test, ANOVA was used, and in case of significance followed by pairwise comparisons (pre vs 1, 3, 6 and 12 months) using t-test.

Furthermore, we investigated the tissue-homing receptor CCR6 since its ligand CCL20 (also known as MIP-3α) is abundantly expressed in the gastrointestinal tract (24), where many innate-like T cells preferentially reside. We quantified the expression of CCR6 on T cells before and after aHSCT. CCR6 expression was reduced on CD8+ T cells after aHSCT, while CD4+ T cells expressed similar levels as healthy controls (**Figure 5A**). Absolute numbers of CD8+ CCR6+ T cells were decreased over a prolonged period of at least two years, while CD4+ CCR6+ T cells dropped only transiently and then recovered (**Figure 5B**).

**Figure 6:**
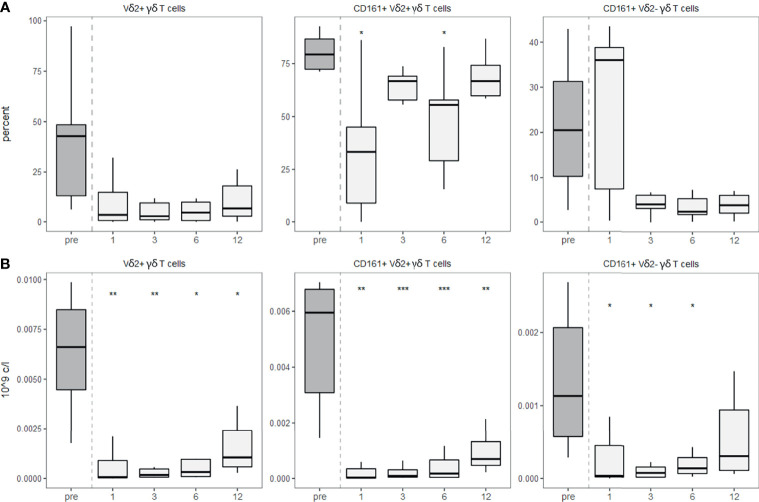
γδ T cells show decreased usage of Vδ2 after aHSCT in MS. PBMCs were stained with fluorophore-conjugated surface antibodies and analyzed by flow cytometry. **(A)** Percentage of Vδ2 fraction of γδ T cells (left), percentage of CD161+ cells within Vδ2+ γδ T cells (middle), percentage of CD161+ cells within Vδ2- γδ T cells (right). **(B)** Absolute numbers of Vδ2+ γδ T cells (right), absolute number of CD161+ Vδ2+ γδ T cells (middle), absolute number of CD161+ Vδ2- γδ T cells. Number of samples were pre = 7, M1 = 7, M3 = 7, M6 = 7, M12 = 7. As a global test, Kruskal-Wallis was used, and in case of significance followed by pairwise comparisons (pre vs 1, 3, 6 and 12 months) using Wilcoxon signed-rank test.

Although γδ T cells could theoretically have a higher diversity than the αβ TCR repertoire (25), the diversity of the γδ TCR repertoire is usually smaller and shows use of invariant TCRs similar to other innate-like T cells including MAIT and NKT cells (26). The most commonly used V gene in the blood of humans is the Vδ2 chain, usually pairing with the Vγ9 (26). By staining for this TCR variable chain, we detected a strongly decreased Vδ2 usage post-aHSCT. While both Vδ2+ and Vδ2- populations expressed less CD161 after the transplantation, this effect was significant in the Vδ2+ cells (**Figure 6A**). As expected, the absolute number of Vδ2+ γδ T cells as well as CD161+ Vδ2+ γδ T cells and CD161+ Vδ2- γδ T cells decreased after aHSCT (**Figure 6B**).

**Figure 7:**
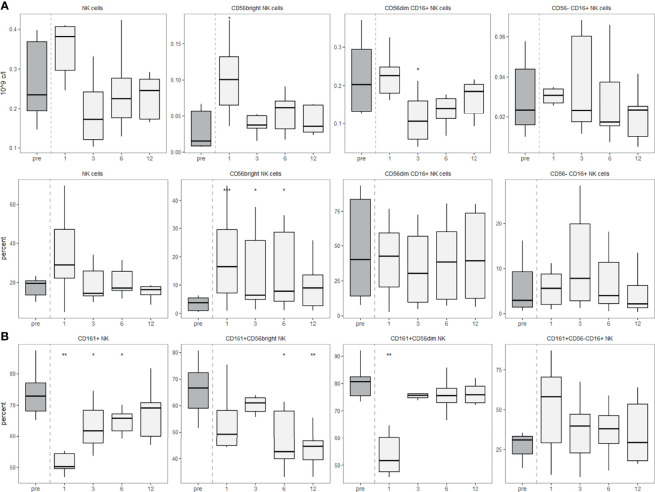
CD56^dim^ NK cells express permanently less surface CD161 after aHSCT in MS. PBMCs were stained with fluorophore-conjugated surface antibodies and analyzed by flow cytometry. **(A)** Absolute (top) and relative (bottom) recovery of NK cells (left, percentage of lymphocytes) and CD56^bright^, CD56^dim^ CD16+ and CD56-CD16+ NK cell subsets (percentage of NK cells). **(B)** Expression of CD161 by all NK cells (left) and their subpopulations. Number of samples were pre = 7, M1 = 7, M3 = 7, M6 = 7, M12 = 7. As a global test, Kruskal-Wallis was used, and in case of significance followed by pairwise comparisons (pre vs 1, 3, 6 and 12 months) using Wilcoxon signed-rank test.

Finally, we also analysed NK cells in more detail by staining for CD16 and the expression of CD161 on the different NK cell subsets. These data largely are in line with the results shown in **Figure 1** (**Figure 7A**). We detected a slight decrease in the percentage of CD56^bright^ NK cells expressing CD161 one year after the aHSCT. Interestingly, one month post-aHSCT, the CD56^dim^ cells showed a significant decrease in CD161 expression, while CD56-CD16+ NK cells did not change at month 12 after transient increases in the prior months (**Figure 7B**).

## Discussion

In this study we describe the immune reconstitution dynamics of NK and innate-like T cells after aHSCT in MS patients. With the help of our broad cell subset panel, we found a strong increase in CD56^bright^ NK cells, while CD56^dim^ NK cells had an overshoot at one month post-aHSCT and then a drop below HC levels. The different functions and roles of the CD56^bright^ and CD56^dim^ subsets have been discussed previously. Some authors stress the role of CD56 as an activation marker (19), while others focus on its differential expression during NK cell differentiation (27, 28). In detail, CD56^bright^ NK cells are thought to be the least differentiated and “youngest” NK cell subset and mount responses mostly, but not entirely *via* producing cytokines. Subsequently, they can mature into CD56^dim^ NK cells, which mediate their lytic activity following NK receptor-mediated activation (29). CD56^bright^ NK cells show immunoregulatory functions like cytokine secretion with low cytotoxicity, while CD56^dim^ cells usually express CD16 (Fcγ receptor III), have predominantly cytotoxic functions and can lyse, amongst others, activated T cells (19, 27, 28, 30). Moreover, there is a NK cell subset lacking CD56 expression while being positive for CD16. These CD56-CD16+ NK cells seem to be similar to the CD56^dim^ NK cells, at least in a proteomics-focussed analysis (31).

The role of NK cells in MS is not clear. A dysregulation of CD56^bright^ NK cells has been reported for MS patients (32, 33). The treatment of MS with the anti-CD25 (IL-2Rα) monoclonal antibody daclizumab identified the expansion of immunoregulatory CD56^bright^ NK cells and the latter effectively blocked disease activity (30, 34, 35). Subsequent studies showed that this cell population is likely affected by other MS treatments as well (30, 36–40). The mechanism(s) of action of CD56^bright^ NK cells in MS includes a dysregulation in their ability to lyse activated, but not resting CD4+ T cells (30, 39). This dysregulation is attributable to an impairment in the DNAM-1 interaction of NK cells with CD155 on T cells (39). IL-2 can stimulate NK cells to lyse T cells, which might be the mechanism by which daclizumab restores this impairment (30, 38, 41). CD56^bright^ NK cells were also reported to be increased after aHSCT in MS (42–44). On the other hand, myelin damage in MS might partially be driven by ADCC *via* Fcγ-Receptors present mostly on CD56^dim^ NK cells (45), which could indicate a pathogenic role of CD56^dim^ NK cells in MS. Furthermore, a previous study observing NK cells in aHSCT in MS demonstrated a negative correlation of NK cells with Th1 and Th17 cells (44). They depleted post-aHSCT PBMCs of CD56+ NK cells *ex vivo*, stimulated them and analysed the Th1 and Th17 response, and reported increased Th1 and Th17 responses in case of NK cell depletion (44), presumably due to NKG2D-mediated killing of T cells in the presence of NK cells (44, 46). Our data shows that CD56^bright^ NK cells are significantly increased, while CD56^dim^ NK cells are stable/decreased except for one month post-aHSCT. Moreover, NK cells were shown to be more sensitive to clinically relevant concentrations of ATG than T cells *in vitro* (47). Therefore, “old” cytotoxic CD56^dim^ NK cells are likely to be depleted by the aHSCT, and afterwards “new” immunomodulatory CD56^bright^ NK cells emerge and predominate, an outcome that might positively influence a pathological NK cell-related disturbance in MS. In one study, the cytotoxicity of NK cells after high dose chemotherapy (BECH or BEAC protocol) was decreased in patients with non-Hodgkins’s lymphoma (48), indicating that this could be part of the immunological effects of aHSCT in MS.

Another interesting population are CD161+ NK cells. Prior data showed that CD161 expression on NK cells is associated with IFNγ production (49) but also with inhibition of cytolytic activity (50), which might allow NK cells to support a proinflammatory environment while reducing their lytic activity towards e.g. autoreactive CD4+ T cells. Our observation of lower expression of CD161 in NK cells post-aHSCT might therefore indicate a beneficial reduction in the secretion of proinflammatory cytokines together with an increased potential to kill autoreactive T cells.

To interpret our results on the innate-like T cells, it is important to consider their functions and properties in healthy individuals as well as their role in MS, or more specifically in the context of aHSCT in MS. Here, we give a very brief profile of the studied innate-like T cells. **Figure 2** attempts to depict the different roles of these cells graphically.

NKT cells preferentially reside in the colon (51) and the liver (52). An important subpopulation of NKT cells carry the invariant TCR chains Vα24-Jα18 and Vβ11 that recognize α-galactosylceramide (αGC) and are referred to as type I, classical NKT or invariant NKT (iNKT) cells. NKT cells with other TCRs are called type II or non-classical (22). Since not only NKT cells but also classical T cells can express CD56 (53), the non-CD1d-restricted CD3+CD56+ double positive population is referred to as NKT-like (22). The name “NKT-like cells” reflects the heterogeneity of this non-CD1d restricted CD56+CD3+ population, containing conventional T cells (e.g. CD56+CD4+ autoreactive and HLA class II-restricted T cells as described by Vergelli and colleagues (20)), MAIT cells and γδ T cells. MAIT cells are commonly defined as T cells expressing the invariant TCR chain Vα7.2 recognizing a riboflavin-derivative presented by a molecule called MHC-related 1 (MR1) (54). As their name already suggests, they are enriched in the mucosa of organs like the gut (54). There, they might be involved in the sensing of microbiota, providing barrier protection and the initiation of tissue repair responses (55). While both NKT as well as MAIT cells express an αβ TCR, there is another T cell subset expressing a γδ TCR. This population is characterized by their early activation following stimulation with conserved stress-induced ligands and is greatly enriched in epithelial tissues like the skin and the gastrointestinal tract (26), but also in the liver (52).

The role of these innate-like T cell populations in MS remains to be studied in more detail. We describe part of the evidence suggesting an involvement in the pathophysiology of MS. Innate-like T cells might directly contribute to the demyelination in the CNS, since the heterogeneous population of CD56+CD4+ T cells but also γδ T cells were reported to lyse oligodendrocytes in MS (56, 57). The local role of innate-like T cells in the CNS is further supported by the presence of MAIT cells (58), and clonally expanded γδ T cells (59, 60) within the CNS of MS patients. Especially the γδ T cells using the Vδ2 chain showed higher expression of CD161 in MS, which was associated with better transendothelial migration (61). Hence, the decreased usage of the Vδ2 chain likely represents the reduction of a γδ T cell subpopulation that seems more prone for CNS infiltration. Moreover, innate-like T cells might contribute to a proinflammatory environment by the secretion of Th1/Th17 cytokines as described for iNKT cells (62) and MAIT cells (63). Notably, iNKT cells are able to recognize a lipid called α-GC, and also polyacetylated GC, a derivative of GC present in the myelin sheath (64, 65). In MS, iNKTs were found to be hyporesponsive towards these myelin antigens (64, 65). The authors hypothesize that this is a consequence of their stimulation and subsequent anergy, possibly as a consequence of prior myelin destruction (66). Hence, most findings suggest that innate-like T cells are probably drivers of the disease and their efficient reduction after the transplantation may contribute to the treatment effects of aHSCT in MS.

There are only limited data about the influence of cyclophosphamide, BEAM or ATG on innate-like T cells. Nevertheless, there is one study that showed a faster recovery of MAIT cells than we describe here after various myeloablative regimens in haematological malignancies (67), indicating that ATG possibly has not only a cytotoxic effect on NK cells but also on MAIT cells.

NKR-P1A, also referred to as CD161, is a homodimer belonging to the C-type lectin family and is expressed by most NK cells (68). CD161 expression on T cells can be inhibitory, stimulatory, or act as a survival signal (23). Furthermore, it has been associated with tissue homing, cytotoxicity and a memory phenotype (23). CD161 expression distinguishes conventional and innate-like T cells, with low expression on conventional T cells and high expression on innate-like T cells. In other words, CD161 has been discussed as a marker of “innateness” (69). Interestingly, MAIT cells greatly overlap with CD161+ CD8+ cells and mostly show a Th17 cytokine profile (70, 71). In MS, CD161 expression on CD8+ T cells has been reported to be increased (72). In line with that, a proinflammatory IL-17-producing CD161+CD8+ T cell population was reported to be decreased after aHSCT in MS (42, 43). The authors mention this reduction of MAIT cells, however, one has to consider that immature MAIT cells mostly do not express CD161 (73). Hence, our observation of a reduced CD161 expression on innate-like T cells post-aHSCT could indicate the normalization of surface CD161 towards a physiological level or even a beneficial decrease below that. Functionally, this might point at a lower CNS-infiltrating capacity, reduced cytotoxicity (e.g. against oligodendrocytes), a more immature or adaptive cell population.

Finally, we studied CCR6 expression on CD3+ T cells and found less CD8+ T cells expressing it post-aHSCT. CCR6+ CD4+ T cells are mostly associated with the Th17 phenotype, while the CCR6+ CD8+ T cell phenotype is less well characterised (74). CCR6 is preferentially expressed by memory T cells (75), and drives mostly the migration of CD8+ T cells and NKT cells towards CCL20, while it is a less potent migration stimulus for CD4+ T cells (53). The decreased surface expression of CCR6 on CD8+ T cells post-aHSCT might point towards a reduced recruitment of *bona fide* CD8+ T cells and CD8+ expressing NKT cells to CCL20 producing tissues like the intestines. Whether the potentially reduced tissue homing to the gastro-intestinal tract (GIT) post-aHSCT is relevant for post-aHSCT outcome remains to be clarified. Furthermore, lymphocyte numbers might already be reduced in the GIT because of the strong reduction in the abundance of innate-like T cells. In this regard it might be also important to take into account the reported microbiota dysregulation in MS (76), though the precise interplay between lymphocytes and microbiota in MS pathophysiology remains to be elucidated in more detail.

Our results indicate that effects beyond the renewal of adaptive immune cells, namely beyond CD4+ and CD8+ T cells and B cells, may contribute to the excellent treatment effects of aHSCT in MS. Regarding the mechanism(s) of action of aHSCT in MS, the increased abundance of regulatory CD56^bright^ NK cells, a long-term reduction of cytotoxic CD56^dim^ NK cells combined with a reduction of proinflammatory innate-like T cell populations appear to play a role. Moreover, phenotypic changes such as a lower surface expression of tissue-homing receptors in innate-like T cells or a diminished usage of the Vδ2 chain in γδ T cells might add to the inhibitory effects on disease activity in MS.

## Data Availability Statement

The raw data supporting the conclusions of this article will be made available by the authors, without undue reservation.

## Ethics Statement

The studies involving human participants were reviewed and approved by Kantonale Ethikkommission Zürich. The patients/participants provided their written informed consent to participate in this study. Written informed consent was obtained from the individual(s) for the publication of any potentially identifiable images or data included in this article.

## Author Contributions

JRu: Conceptualization, establishment of techniques, performing experiments, data curation, data analysis and interpretation, writing - first draft, revision and editing. JRe: Isolation of biomaterials, editing. SO: Data acquisition, editing. MD: Establishment of techniques, editing. AM: Investigation, clinical aspects, editing. US: Investigation, clinical aspects, editing. IJ: Investigation, clinical aspects, editing. RM: Conceptualization, data interpretation, funding acquisition, validation, writing – review and editing. All authors contributed to the article and approved the submitted version.

## Funding

This study was supported by a Swiss National Science Foundation (SNF) grant (323530_183985) for MD/PhD students awarded to JRu, and SNF grant (32003B_185003) to RM.

## Conflict of Interest

RM received unrestricted grant support from Biogen, Novartis, Hoffman La Roche and Third Rock, and compensation for advice or lecturing by Biogen, Novartis, Sanofi Genzyme, Merck, Hoffmann La Roche, Neuway, CellProtect, and Abata. RM is employed part-time by Cellerys, a startup company outfounded from the University of Zurich. He is a co-founder and stockholder of Cellerys and a co-founder of Abata Therapeutics. RM is listed as an inventor on patents of the University of Zurich about target antigens in multiple sclerosis. RM is further listed as inventor and received remuneration for a NIH-held patent on the use of daclizumab to treat multiple sclerosis. None of which has impact on the submitted work.

The remaining authors declare that the research was conducted in the absence of any commercial or financial relationships that could be construed as a potential conflict of interest.

## Publisher’s Note

All claims expressed in this article are solely those of the authors and do not necessarily represent those of their affiliated organizations, or those of the publisher, the editors and the reviewers. Any product that may be evaluated in this article, or claim that may be made by its manufacturer, is not guaranteed or endorsed by the publisher.
