# Supplementary material for: NK Cells and Innate-Like T Cells After Autologous Hematopoietic Stem Cell Transplantation in Multiple Sclerosis
Source: Front Immunol. 2021 Dec 16;12:794077. doi: 10.3389/fimmu.2021.794077 (PMC8716406; doi:10.3389/fimmu.2021.794077)
Supplement: Supplementary file 1 [file DataSheet_1.docx]

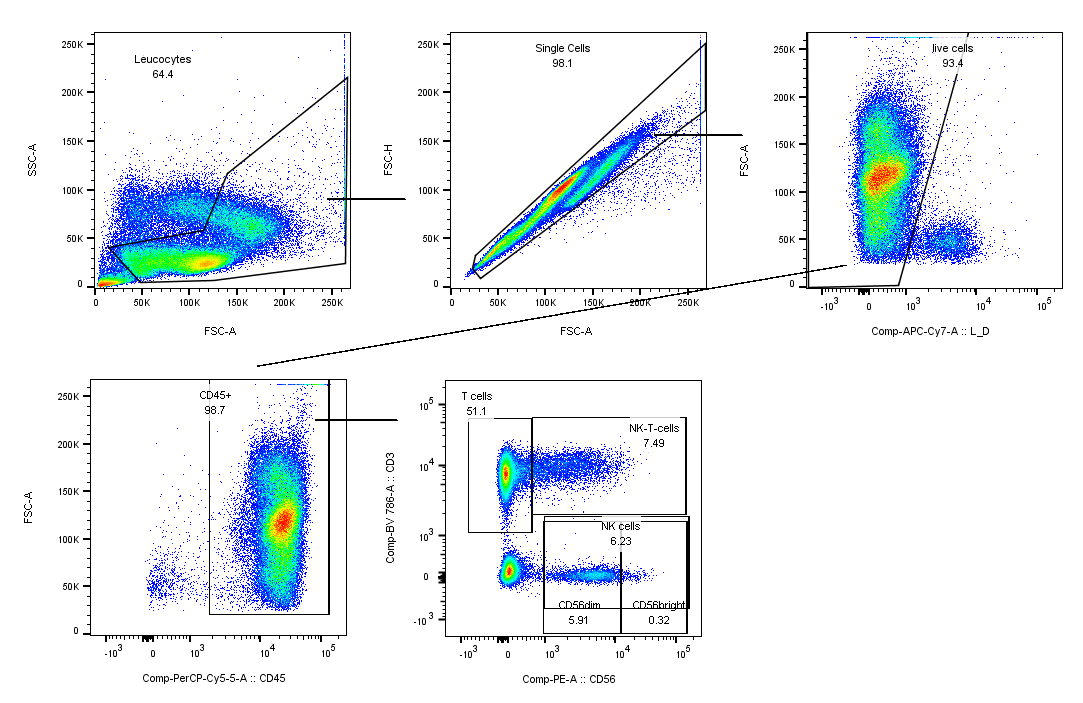


**Supplementary Figure 1**: Gating strategy of the broad cell subset panel to characterize NK cells and T cells. Leucocytes, single cells, live cells and CD45+ cells were selected, and further characterized as *bona fide* T cells, innate-like T cells and NK cells.


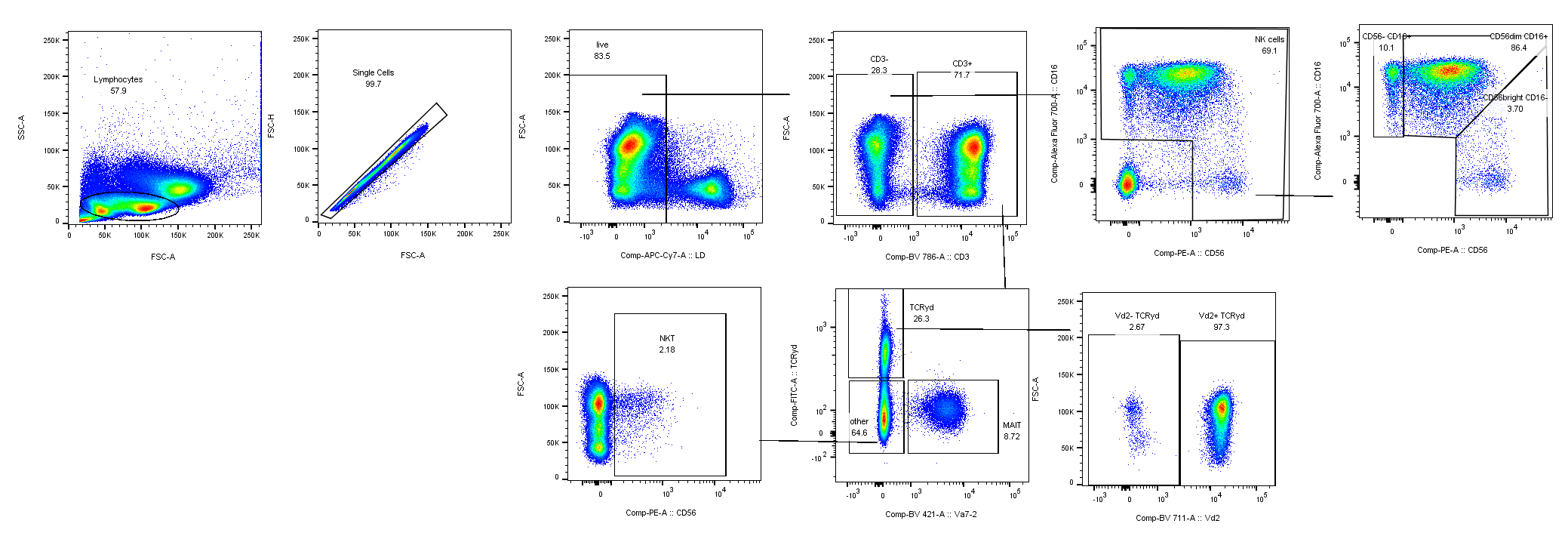


**Supplementary Figure 2**: Gating strategy of NK and innate-like T cell panel. First, lymphocytes were selected, followed by single cells and live cells. Next, CD3+ T cells or CD3- non-T cells were gated. From non-T cells, gates for NK cell subsets were drawn. From CD3+ T cells, $\gamma\delta$ T cells and MAIT cells were selected by their T cell receptor (TCR). NKT cells were defined as negative for the Vα7.2 and $\gamma\delta$ TCR and positive for CD56 as a NK-associated receptor.


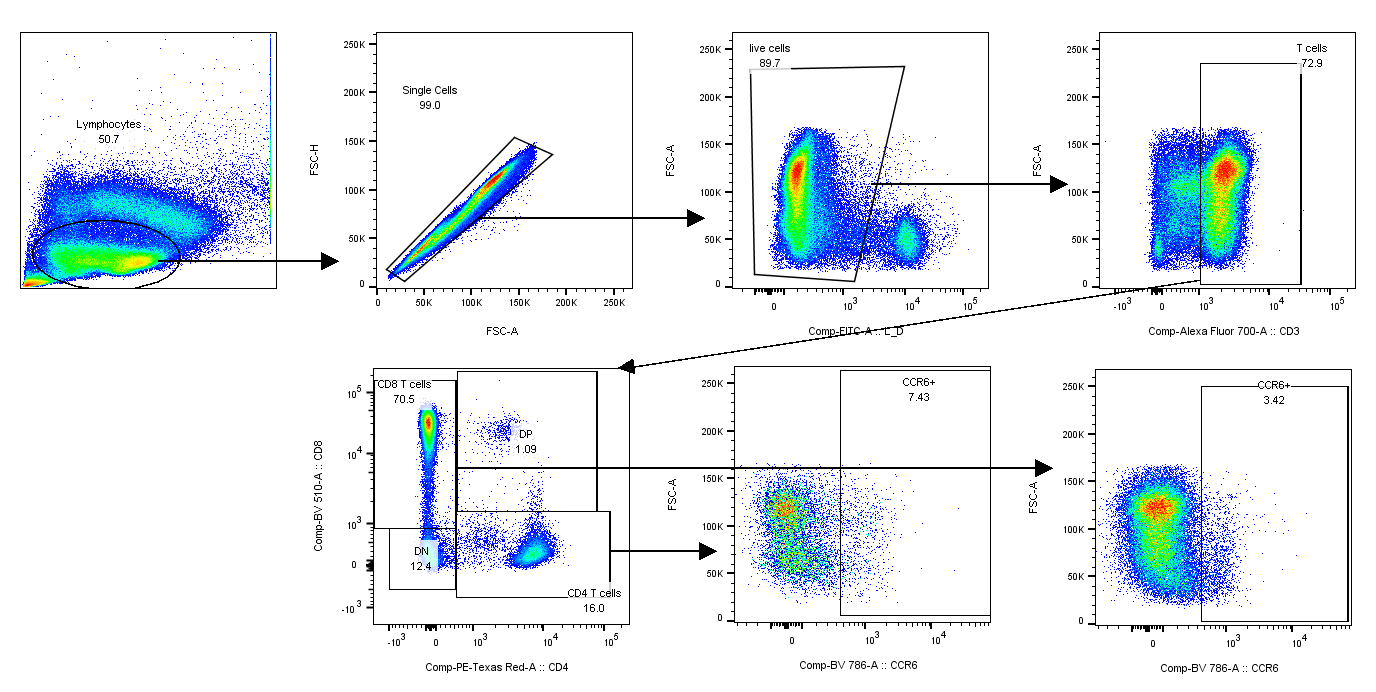


S**upplementary Figure 3**: Gating strategy of the CCR6 panel shows first the selection of lymphocytes, single cells and live cells. After CD3+ T cells were gated, we differentiated between CD4+ and CD8+ T cells. On these two populations, the percentage of cells expressing CCR6 was determined.


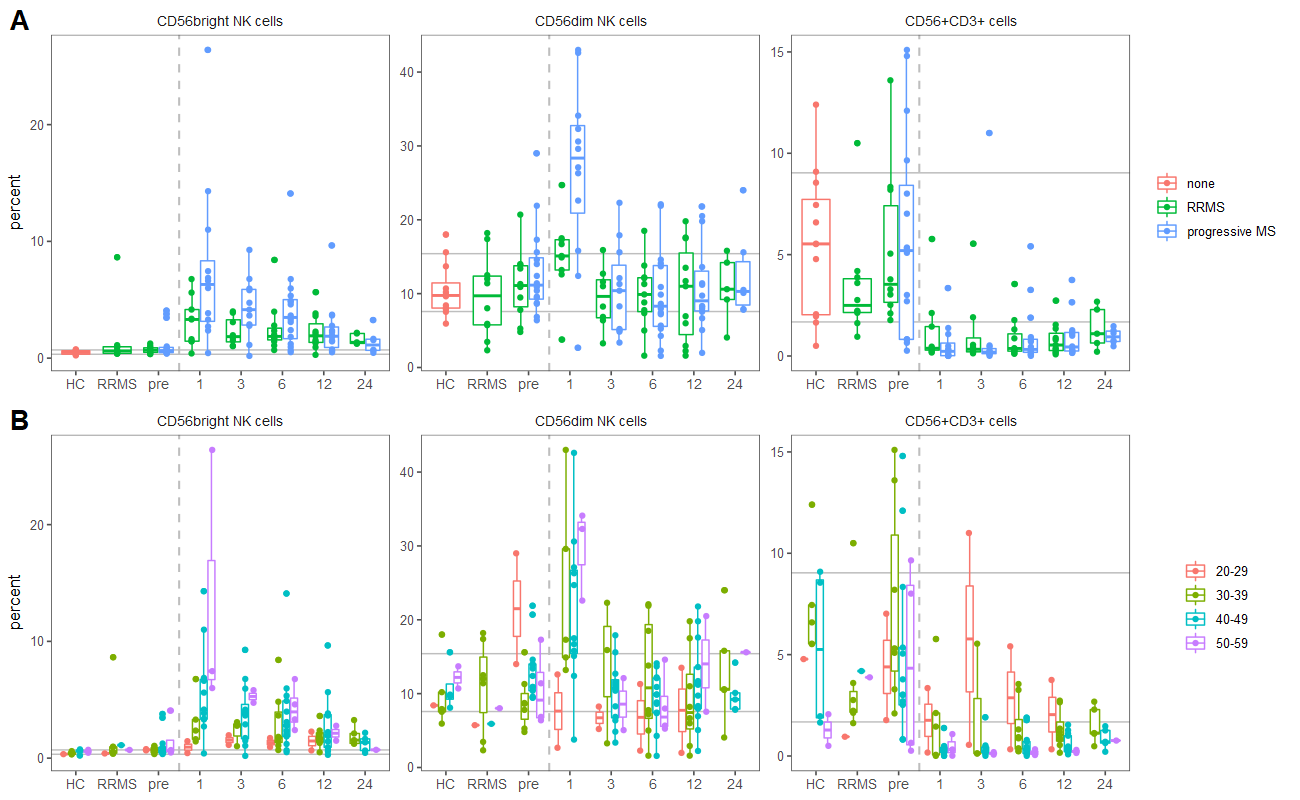


**Supplementary Figure 4**: **Age and disease type seem to have an effect on NK and CD56+CD3+ T cell reconstitution.** PBMCs were stained with surface fluorophore-conjugated antibodies and analyzed by flow cytometry. Percentages are fractions of total PBMCs. (A) Reconstitution of CD56bright (left), CD56dim (middle) NK cells innate-like CD56+CD3+ T cells (right) depending on the diagnosis. (B) Reconstitution of CD56bright (left), CD56dim (middle) NK cells innate-like CD56+CD3+ T cells (right) depending on the age of the patients. Horizontal lines reflect the 10th/90th percentile of age- and sex-matched HCs. Number of samples were pre = 26, M1 = 21, M3 = 19, M6 = 27, M12 = 25, M24 = 11, HC = 12, RRMS = 10.
